# Nurses’ experiences of text-based digital triage at primary healthcare centres in Sweden: a qualitative interview study

**DOI:** 10.1186/s12912-025-02683-z

**Published:** 2025-01-14

**Authors:** Ester Rydell, Ulf Jakobsson, Sigrid Stjernswärd

**Affiliations:** 1 Department of Health Sciences, Faculty of Medicine, Lund University, Lund, Sweden; 2 Department of Clinical Sciences (Malmö), Center for Primary Health Care Research, Faculty of Medicine, Lund University, Lund, Sweden

**Keywords:** Communication, Text-based digital Technology, Experiences, Literacy, Nurse, Primary Healthcare, Telehealth, Triage

## Abstract

**Background:**

Telehealth services are becoming increasingly popular at primary healthcare centres. Some examples include text-based digital triage and health guidance using chats, emails, images and pre-filled forms. Telephone-based communication has until recent years been the predominant means for triage and health guidance, but now includes written communication via computer or smartphone. Hence conditions to perform triage and health guidance have changed, which may affect the quality of nurses’ work and patient safety. This motivates an in-depth exploration of the consequences of such changes for nurses working with telehealth. The study aimed to explore nurses’ experiences of digital triage and health guidance at primary healthcare centres in Sweden.

**Methods:**

A qualitative inductive design was chosen for the current study. Four registered nurses and two public health nurses, working at primary healthcare centres in southern Sweden, contributed with information about text-based digital triage and health guidance. Semi-structured interviews were conducted to collect data. Data were analysed using qualitative content analysis.

**Findings:**

One main theme, “Adapting to a new professional toolbox to triage and give health guidance” was constructed based on three categories which describe the altered professional tools. They were: “Using one’s senses differently to collect information”, “Change of communication mode to convey messages” and “Change of timeframe and the use of time”. Six subcategories describe how the new toolbox was experienced by the nurses. They were: “Loss of clinical ear”, “Gain of viewing images”, “Difficulties in written communication of care and emotions”, “Seeing oneself as a writer or talker”, “Tardy asynchronous communication” and “Available time”.

**Conclusion:**

The findings tell of a substantial change in nurses’ professional toolbox that demanded other skills than they were used to working with. Due to limitations in communication and communication skills, digital triage alone may lead to an impaired workflow, quality of care, and patient safety as well as maintain the digital divide. However, digital triage may also enhance nurses’ work with the addition of attached images, convenient communication for those who are comfortable with writing, and a gain of time for consultation and reflection. The current study contributes insights regarding new competencies that nurses and patients must have or gain to be able to benefit from the possibilities of digitisation of primary healthcare.

## Background

Telehealth is expanding exponentially within healthcare, entailing new technologies, new mindsets as well as new ways of working [1] (p. 1). Text-based digital triage and health guidance using chats, emails, images and pre-filled forms at primary healthcare centres (PHC) in primary care is one example of telehealth development that is becoming more popular internationally [2–4]. Telephone-based communication has until recent years been the predominant means for triage but now includes written communication via computer or smartphone. The implementation of such systems in healthcare can affect healthcare professionals’ clinical routines and ways of working, and patients’ experiences of healthcare and their encounters with, e.g., nurses [2, 5] which motivate the exploration of the consequences of such implementation initiatives.

Telehealth refers to the remote provision of healthcare using telecommunication technology, which includes the use of telephones, smartphones and computers, along with other technical devices [6] as well as “a way of thinking, an attitude” [1] (p. 1). Telehealth nursing is more specifically related to the provision of remote nursing. For a long time, policymakers and the World Health Organization (WHO) have been promoting telehealth as a solution for enhanced healthcare efficiency, quality and equality [7]. This is because it has been proven to decrease healthcare costs and increase clinical efficiency [8, 9] as well as facilitate the provision of care in rural areas [7]. On the other hand, cost-effectiveness is not seen in all health specialties [8]. Furthermore, there are indications of a digital divide leading to the exclusion of those without internet access or, for different reasons, people with low ability to use the internet, i.e. individuals with poor digital literacy [10]. This suggests a risk of deteriorated equality of care. Remote consultation may also risk patient safety by poorer rapport building and inadequate information gathering, limited clinical assessment as well as missed, inaccurate or delayed diagnoses and treatment [11]. Patients have also reported doubtfulness towards the quality of care given online [12].

Internationally, online services such as E-consult (UK), Babylon Health (UK, US, Canada, Rwanda), Doctrin (Sweden), and Kry (Sweden, UK, Norway, France, Germany) are examples of telehealth services within private primary care that provide distance consultations with healthcare professionals. NHS-app (UK), Sundhed (Denmark), and 1177 Health Guide and Online Services (1177 Vårdguidens e-tjänster, Sweden) are examples of nationally owned telehealth services, with similar or simpler functions such as e-mailing, online booking, cancellation of appointments, and requests for prescriptions. Prior to a chat or video consultation with healthcare professionals, health-seeking persons (HSP) are often requested to fill out standardised written forms, which include questions aimed at collecting information relating to the HSP’s reason for contacting the PHC. Direct consultation with a doctor over the telephone, video, e-mail or online chat is popular among patients due to increased availability and the convenience of staying at home whilst getting access to a consultation [13].

Nurses, using telehealth in Sweden, usually perform the initial triage and health guidance using the online services mentioned above. It includes reading the pre-filled forms and messages to assess the HSP’s need for health guidance and/or visits or contact with a specific healthcare professional. The communication between HSP and nurses is text-based, and either synchronous or asynchronous. Text-based digital triage and health guidance in primary care will henceforth be abbreviated with the term “digital triage”. It includes synchronous and asynchronous chat, pre-filled e-forms, e-mail and attaching images, but excludes video consultation.

Although existing research on the subject is scarce, prior studies have indicated clear benefits regarding the use of chat- services including pre-filled forms and images, to deal with simpler health issues [2–4, 14, 15], or with health concerns with distinct visual features including viewing images [16]. Text-based digital communication between health workers and HSPs, who have already established contact, has also been reported as being favourable, for instance for follow-ups after hospitalisation [17, 18]. It can also facilitate continuity of care between patients and healthcare professionals when there is already an existing contact [3, 8, 19]. Patients report overall satisfaction with digital triage in Johansson et al.’s [15] pilot study [12], but they also expressed concerns about the doctor’s ability to assess care needs from a distance. Furthermore, the experience of the communication was rated as “fairly good”.

Digital triage performed by nurses in primary health care, however, has not been studied significantly. Johansson and Ivarsson´s [14] pilot study, which used questionnaires, found that nurses expressed general satisfaction with digital triage. In addition, Johansson et al. [12] performed a similar study from a patient perspective and found comparable results. However, the authors of both studies did not go into detail concerning communication or assessments as such and it was tested on a limited number of simpler health concerns. According to Eldh et al. [2], the implementation of digital triage at PHC involves enacting changes in healthcare professionals’ working routines and approaches to the HSP. Eldh et al. [2] showed that healthcare professionals experienced a loss of important information via the digital service—such as the HSP´s voice, which can reveal signs of symptoms. The overall experience was that of an improved assessment and better use of resources. However, the study included general practitioners (GP), nurses, and other healthcare workers, not only focusing on nurses’ experiences. Both studies implied the need for further research.

Telephone-based triage and health guidance put high demands on communication quality, i.e., nurse's communication skills [20] and the callers’ ability to express needs and symptoms [21] to compensate for the absence of physical examinations and visual cues. The implementation of digital triage implies an altered way of working for nurses as oral communication over the telephone is exchanged by written communication via computer/smartphone. The exchange of communication modes may entail a risk to the quality of care, as experienced by both patients [12], and nurses [22] as well as counteract equality of care [10] and risk patient safety [11]. Existing studies on the topic often merge telephone with text-based digital communication as “telehealth” without distinguishing the different types of communication modalities [23–25]. Qualitative interview studies are therefore motivated to investigate the consequences of such changes.

Hence, the current study’s aim was to explore nurses’ experiences of digital triage, with the objective of gaining new information and perspectives on how it affects their work experiences.

## Methods

A qualitative inductive design was chosen for the current study to explore nurses´ experiences of digital triage with HSPs in primary care. This design permits a flexible and adjustable approach in a rather uncharted terrain as new information is gained, with the purpose of understanding the phenomenon at stake [26]. To ensure transparency of the study’s execution the COREQ checklist [27] was followed.

### Setting and participants

The current study was carried out at Swedish primary healthcare centres. The inclusion criteria were as follows: Working as a registered nurse (RN) and/or a public health nurse (PHN) in a PHC, regardless of age, sex and nationality; having at least one year’s experience of triage and health guidance; speaking and understanding Swedish. RNs and PHNs working in PHC with triage and health guidance, including telephone and digital triage, were thus purposefully sampled as they were expected to have experience from the researched area [28]. PHNs in Sweden are RNs with further academic education and specialisation within care, medical sciences, public welfare and care pedagogics [29]. Three managers at three different PHC were contacted by email. All agreed to participate in the study by allowing the recruitment of nurses through their respective organisations. The PHC were strategically chosen as they could contribute to a diversity of experiences relative to location and type of PHC (see Table 1). One nurse at every PHC was asked to select participants who fulfilled the inclusion criteria for participation. None of the participants were acquainted with the interviewer prior to the interviews. Henceforward, both RNs and PHNs are referred to as nurses in the current paper. RNs and PHNs performed the same tasks related to digital triage, which is why no distinction was made between them when analysing data.

**Table 1:** Participants characteristics

	Public PHC	Private PHC	Total
Number asked to participate:	2	5	7
Final participants:	2	4	6
Age (years):	40–64	40–41	40–64
Sex (F/M):	2/0	3/1	4/1
Profession:	2 PHN	4 RN	2 PHN/ 4 RN
Work experience (years):	18–29 years	1–14 years	1–29 years
Using digital triage and chat service:	0	4	4
Using 1177 health guide and online service	2	4	6
Number of nurses working in rural location	0	3	3
Number of nurses working in urban location	2	1	3

### Data collection

Data were collected using a semi-structured interview guide specifically constructed for the current study (see Table 2) to explore digital triage as it is experienced from the nurses’ perspective. Individual interviews were used with the aim of producing new knowledge through descriptions and interpretations of the phenomenon in question [30]. The interview guide had a wider perspective on experiences of digital health services than the current study’s aim. Open-ended questions were asked about the participants’ experiences of digital health services that aimed to tap into subjects such as quality, efficiency, patient meetings, availability, work environment and administration/journaling. The current study narrowed down its analysis to data concerning experiences of digital triage as the empirical material encompassed rich data on this specific topic. The following questions were asked during the interview, to clarify statements and to reach a deeper understanding of the nurses’ experiences. The interviews were carried out in 2019–2020 by the first author (E.R.) and lasted between 29 to 49 min (mean = 38 min). Five interviews were carried out face-to-face at the participants’ workplace and one was done over the phone. All the interviews were recorded and transcribed verbatim. At the time of the study, the interviewer was working as a PHN at a PHC with similar digital healthcare services, but not in any of the PHC involved in the current study.

**Table 2:** Interview guide

1. Can you tell me about your thoughts and experiences regarding digital health services?
2. What are the advantages and drawbacks?
3. Can you give me examples?
4. Tap into the following questions: ° Quality °Efficiency °Patient meeting °Availability °Work environment °Administration/journaling

### Data analysis

The transcribed interviews were analysed inductively using qualitative content analysis with the aim to unconditionally seek patterns in the collected data to underpin a systematic organisation of the material [28, 31, 32]. The transcribed interviews were read over repeatedly by the first author to get a thorough understanding of the whole material. Meaning units, such as words, sentences, and/or whole paragraphs answering the study’s aim, i.e., nurses’ experiences of digital triage, were identified and extracted from the text. Thereafter, meaning units were condensed and coded. Codes were used to briefly describe the content of the meaning unit and should always be understood from the context of the study [28]. Codes were thereafter grouped into subcategories, which were created by comparing and sorting codes based on their similarities and differences. Coding and subcategorising were performed on a manifest level [28]. This step was reworked several times together with the two other authors (U.J., S.S.) until consensus was reached; this was to make sure that the constructed subcategories reflected the data and that important data answering the study’s aim were not left out. The subcategories were then grouped into three categories with common contents. These categories shed light on the material with a moderate level of abstraction and structured specific issues into clusters [31]. The next step consisted of interpreting the underlying meaning of the categories and subcategories and creating a theme, as a way to connect categories by finding a recurring underlying meaning in the collected data. The analytical process was not a straight line forward, but rather a process of going back and forth between the different steps to adjust the interpretation of data and final findings [31].

### Ethics approval and consent to participate

The study was carried out in accordance with the ethical principles of the World Medical Association [33]. Approval to conduct the study was granted by the heads of the healthcare centres and the nurses’ employers. All participants received written and oral information about the study and the interviewer, including information about their participation being voluntary and that they could withdraw at any time without stating a reason. All participants signed an informed consent form prior to the interviews. The collected data and personal information were handled confidentially, to protect the participants’ identity and integrity. The study did not include any interventions or experiments on humans, nor the collection of so-called sensitive personal data, which is why ethical approval was not needed according to Swedish legislation [34].

### Findings

The nurses experienced that digital triage differed from telephone and face-to-face triage. Digital triage naturally postulates a change from, listening to reading and from speaking to writing. For the nurses, this shift was not only a change of means to work with but meant a considerable change in their “professional toolbox” which affected their abilities to perform their work and had an impact on their workflow. The “Professional toolbox” is a way of describing senses, means and circumstances that the nurses take into consideration to assess, triage and communicate with the HSP, as described in the current findings. It includes physical senses such as seeing, touching, feeling and listening in order to collect information needed for assessments. It also includes physical means such as body language, facial expression, speaking and writing to convey their message to the HSP. Lastly, the time dimension within which nurses have to complete their work stood out as a circumstance that the nurses expressed as important and affecting their workflow and ability to triage. To have professional tools that are innate to the nurses and that can be used without difficulty was perceived as a prerequisite to performing fluent triage. The changed toolbox could affect their sense of comfort and ease with the task at hand as well as their work efficiency. This permeable experience thus reflects the main theme, “Adapting to a new professional toolbox to triage and give health guidance”. The categories describe the change of tools that digital triage has entailed. The subcategories describe nurses’ different experiences concerning the new tools in the professional toolbox (Table 3).

**Table 3:** Scheme of constructed theme, categories and sub-categories

**Theme**	***Adapting to a new professional toolbox to triage and give health guidance***					
**Category**	*Using one’s senses differently to collect information*		*Change of communication mode to convey messages*		*Change of timeframe and the use of time*	
**Subcategory**	*Loss of clinical ear*	*Gain of viewing images*	*Difficulties in written communication of care and emotions*	*Seeing oneself as a writer or talker*	*Tardy asynchronous communication*	*Available time and increased accessibility*

### Using one’s senses differently to collect information

According to nurses, digital triage was associated with both advantages and drawbacks compared to telephone-based communication. Digital triage made use of activities such as reading messages and pre-filled forms with health information as well as viewing images of wounds and rashes, thereby appealing to different senses when performing health assessments. The nurses described a loss of their “clinical ear” which was experienced as an essential tool in telephone- based triage. On the other hand, they gained a (distant) eye, which was useful, but within a more limited area.

#### Loss of clinical ear

The most prominent experience described by the nurses related to the change of senses used to collect information (from listening to reading). Gathering information from telephone contact was already described as difficult due to the limited possibility of examining and seeing the patient. Listening was described as a tool that gave more information about patients’ health status than the digital pre-filled forms and, by patients, written messages. Parameters such as breathing, coughing, expressions of pain and chest pain, mental state, emotions, tonality and personal traits were mentioned as examples of important information that are available in telephone contact, but not through digital interaction. The act of listening was thus also a way of trying to grasp the patients’ situation even beyond their own words, to facilitate assessments of the HSP’s health condition and needs. Digital triage was experienced as taking a step farther away from the patient as one loses what can be interpreted as the “clinical ear”.

*“Well, it’s easier to listen. Actually, we work a lot with that in healthcare, when, with both what you hear and see. So, telephone is one thing, not being able to see the patient, that makes it harder. And to talk, so to say, *via* the computer takes it a further step away, because then you can’t even hear.” (3).*

The nurses found it more difficult to interpret the patient’s needs with digital triage when they lacked the clinical ear. Lacking the clinical ear could lead to difficulties and faulty levels of care when deciding whether patients should be referred for further treatment. For instance, it was difficult for nurses to estimate what the patient meant with specific answers in the prefilled forms and free text messages. At times, the information could even be interpreted as misleading due to different interpretations of this information by the nurses compared to the HSP intention and/or interpretation. One included a nurse’s assessment of the HSP’s description of abdominal pain, which was not on par with its level of seriousness. The HSP’s written description of abdominal pain led the nurse to book an emergency appointment for the same day. However, the HSP replied several days later and thereby missed the appointment. This indicates that the HSP had another interpretation of the severity of the condition but expressed it in a way that it was interpreted as being more severe. This speaks for the differing meanings and interpretations of words. In telephone triage, when using the “clinical ear”, nurses felt that they could get around the varying ways for HSP to present their needs and symptoms, based on verbal and nonverbal communication, and make a sound assessment of their health condition and needs. However, with digital triage, nurses felt that they had to compensate for the loss of the clinical ear, by untangling uncertainties, using more questions, starting a written dialogue, and at times even phoning the patient. Another solution was to book appointments in the absence of a confident digital assessment.

*“It’s a little like, everybody that has a nasal congestion fills in “breathing difficulties”… and to me that means something else than nasal congestion so, often it can get a little slow, you kind of have to deal with, ok, let’s start with your breathing, “how is your breathing?” ok, then that’s completed. And then they also fill in chest pain, so it can get a lot in those standardised forms that gets, eh, misleading in a way, so it becomes time-consuming.” (1).*

#### Gain of viewing images

According to the nurses, digital triage was associated with some advantages, such as efficient, remote care and diminished risks of contagion. For instance, within the health areas with visible symptoms such as shallow wounds, suspected skin infections, rashes, dots and eczema, errands were experienced as efficient to handle as compared to telephone-based interactions. With digital triage systems, which included the transfer of images, HSPs could thus get adequate help from the comfort of their homes. Nurses could look at the attached images, assess the patient’s condition, give advice and book a physical meeting or follow-up through the chat if needed. In addition, patients with highly contagious conditions, such as suspected smallpox, could keep away from visiting the PHC and thus avoid exposing others to risks of contagion.

*“Sometimes it can even be the case on the telephone, that they have a rash but are unable to describe it. So, we refer it to the chat, send the link by phone, and in short, we can see what it is. This looks like smallpox, or chickenpox, they don’t even need to come here. And chickenpox shouldn’t even come here. So that’s great, *via* the chat.” (6).*

Whether used or not, nurses referred to functions in digital triage that could be useful, indicating that they, in some respect, gave thought to these technical solutions, but that they, for some reason, had not made use of them. For instance, they did not use video contact, even though they often referred to it as a possibility, which offered a combination of using both the clinical ear and eye. Despite this possibility, however, nurses felt hesitant to use it as they reported feeling uncomfortable showing themselves on video.

### Change of communication mode to convey messages

The former category dealt with the means to receive and interpret impressions on different levels, whereas the following category explains the equally multifaceted task of communicating back to the patient. Communicating with HSP involves the transfer of information and assessments as well as showing empathy and emotions and taps into nurses’ potential disposition as a “writer” or “talker”.

#### Difficulties in written communication of care and emotions

In comparison with telephone contact, nurses described digital written dialogues as emotionally more distant and impersonal. It was experienced as more difficult to express empathy and sympathy in writing. The communication of emotions was deemed important and central to supporting personal encounters with HSPs, and nurses estimated that their voice, including tonality, was important to convey both empathy and sympathy. For example, nurses reported restrictions in terms of mediating sympathy through their voices when using written communication whilst having to deny HSP wishes for a doctor's appointment, e.g., due to limited resources or triage decisions that HSPs did not agree on. Nurses also worried about HSPs’ interpretation of the written word differing from their intention. For instance, they worried about sounding disagreeable or judgmental in text messages.

*“I have become quite anonymous with the digital service. I can’t hear how they actually are, what is up… with telephone it’s a personal meeting, even though it’s just a conversation. That, I believe, gets lost in the digital world….. And it also has to do with tonality, it’s possible to ask them “how are you, really?” How does one write that, without sounding judgmental?” (2).*

However, nurses could, at times, appreciate the physical distance incorporated in digital triage, for instance when patients’ requests or communicative style were experienced as difficult to deal with. In such cases, the distance was described as a relief. For example, when they had to deny a prescription for addictive medication.

#### Seeing oneself as a writer or talker

Another important aspect addressed by the nurses was the ability to express themselves in a way that felt natural and straightforward. Some nurses felt reluctant regarding digital interactions because of the requirements to communicate through writing. They described themselves as “talkers” rather than “chatters/writers” and described difficulties in expressing themselves in written form. This limited them in their work and workflow since an important professional tool was lost. In contrast, other nurses appreciated digital interactions because of the written communication. They enjoyed sinking into the process of writing and what they experienced as a quieter work environment. It also gave them a chance to give the HSP more detailed information, including links to relevant websites.

“I think it’s me personally that finds it a little bit difficult to express myself. It’s easier for me when I speak. I don’t have that problem when I talk on the phone.” (3)

Nurses not only viewed themselves as mainly writers or talkers but also perceived similar tendencies in patients. This could either facilitate or hinder their work. While some HSPs were experienced as easy to communicate with through text messages as they were able to express themselves clearly, other patients’ communication and needs were difficult to grasp. At times, nurses experienced that it was difficult to get relevant information from some patients—no matter how many questions they asked. In that sense, the requirement for written communication imposed by digital triage and guidance could challenge nurses’ ability to provide adequate help to patients.

“It depends a lot on the patients, they must know what they’re getting into—text communication. Not all do, sometimes one really must drag the words out of them.” (1)

### Change of timeframe and the use of time

Nurses experienced that digital triage engendered changes in the timeframe within which they were used to work in contradictory ways; they experienced a tardy, time-consuming work process, but also a feeling of having gained more time for reflection and consultation.

#### Tardy asynchronous communication

Nurses reported that, through telephone contact, there is synchronous communication with one HSP at a time. Assessments are made and decisions executed within a limited amount of time. On the one hand, access to information from the patient, using the clinical ear as well as synchronous communication, is direct. On the other hand, time is limited to triage and caregiving. Digital interaction changes communication towards a non-direct, unlimited exchange of written information prolonging the contact if the different parties allow it to. Chatting and writing asynchronously were seen as time-consuming due to the respective modalities’ inherent characteristics and due to the process of typing text itself. Replies did mostly not come directly but could linger. Some chat conversations could go on for days before being resumed. In addition, because of the unrestricted way of communicating, text messages were at times experienced as lengthy.

*“One completes a telephone contact much faster…this (digital triage) can go on all day long and sometimes for several days, those errands. Per visit, so to say, it surely isn’t time efficient.” (2).*

Another consequence of the tardy communication and unrestricted time frame was a feeling of uncertainty in terms of managing more acute, severe or complicated errands. Chatting and writing messages imply that the nurses cannot know when the HSP will read their replies. Nurses experienced that it was unsafe to deal with symptoms such as chest pain, suicide risk and acute infections for that reason. This is why they sometimes chose to call the HSP to make sure that their message came through. To call instead of continuing a written dialogue was also a “work around” to be more efficient when the errand was complicated and needed thorough explanations.

*“Yes, all suspicions of suicide risk, because that can come, it’s not unusual to get [*via* the digital service]. We call them, of course. Otherwise, we often ask them to call instead.” (4).*

#### Available time and increased accessibility

A clearly positive consequence of the asynchronous communication and unrestricted time frame was an experience of freed-up time for reflection, consultation and support as well as a chance to gather information. In the context of telephone contact, assessments and decisions must be carried out within the time frame of the phone call or be momentarily ended for a later contact or follow-up. Correspondingly, the HSP had the same advantage of freed-up time, as seen from the nurses’ perspective.

*“The benefit of a chat is that the patients can take their time to think, I mean if we ask questions, they don’t need to answer at once, which means that they can gather a lot more information.” (6).*

Nurses also experienced that digital services increased access to healthcare, saving HSP time and making their contact with PHC more convenient. Accessibility to PHC was also seen to increase through “open errands”, a contact that is initiated but on hold. For example, nurses gave HSPs advice and, depending on the development of the patient’s health status, a follow-up might be needed if the patient’s condition did not improve. Errands thus remained on the digital service (on hold) but could be easily activated by sending a message through the chat, with all the necessary information being readily available. Compared with the telephone, which requires HSP to call again, wait in a queue, and perhaps explain their errand again to a new nurse, it was a great benefit. The nurses believed that this was appreciated by the patients in terms of increasing accessibility and creating a feeling of security.

*“A clear advantage for the patients is that it provides a kind of security. We can always keep an errand; we’ve handled some errands by letting them stay open. Write again if you feel worse tomorrow” (2).*

## Discussion

The main finding of the study is that the changes in communication, from listening to reading and from speaking to writing, challenge the nurses’ habitual toolbox and require an adaptation to a new set of tools. Even though the use of digital triage pointed to some advantages, the current findings show that digital triage leads to the loss of the “clinical ear”, which perhaps is the most important professional tool that nurses make use of in telehealth to collect information underlying their assessments for triage. It also deprived some of the nurses of their ability to give care and maintain a fluid workflow as they did not feel comfortable with writing. The discussion section will focus on possible drawbacks and advantages, of digital triage concerning triage assessment quality, as well as possible consequences of the shifted means of communication, and its implications.

The nurses in the current study experienced that telephone triage as compared to a physical encounter was a challenge, but they also described how they relied on their “clinical ear” in telephone triage. Earlier studies similarly describe how nurses performing triage over the telephone have developed other means to compensate for the absence of physical encounters to collect information underlying their assessments by listening to more than just words [35–37] reinforcing the “clinical ear” as a significant professional tool for telehealth nursing assessments. It means that nurses listen to the calling patients' way of presenting information, encompassing tonality, behaviour, surrounding sounds, and what is indicated but not said, thus including both verbal and nonverbal communication [35]. It has also been referred to as “listening to an inner voice” while paying attention to clues and signs [37, 38]. To acquire clues and signs, nurses have reported how they, for example, guide calling patients towards self-examination [38]. Replacing the clinical ear with text-based communication may thus impair the quality of triage assessment due to more limited possibilities to collect information as compared to, e.g., physical meetings or telephone triage. Impaired triage quality may lead to unnecessary healthcare appointments [39] or the inadvertence of potentially serious health conditions [3].

In addition, the current findings, just like Enterzarjou et al.’s [3] study, report about pre-filled forms and messages that led to irrelevant information in relation to patients’ health concerns, which nonetheless had to be dealt with and thereby consumed time and energy. Fixed checklists and structures are reported in a review study [40] to restrict HSP provision of a detailed description of the situation. HSP reported feelings of irritation due to numerous irrelevant questions and also reported that they experienced the service as impersonal. Misinterpretation of the text from both patients and healthcare professionals has been reported as another risk [3, 19] and is aligned with the current findings. This demonstrates that the loss of the clinical ear gave rise to a need to pose follow-up questions to verify, clarify and fill information gaps. A likely shortcoming of digital written triage in comparison to oral communication, except for the additional time that such communication can imply, is hence the difficulty to ‘capture the whole picture’, as feared by the contributors in Öberg’s study [22]. Patients particularly at risk in remote consultation, due to limited clinical information, as described by Payne et al. [11] are those with complex, multiple and pre-existing conditions, cardiac-, or abdominal emergencies, vague or generalised symptoms as well as those who had difficulty communicating. Thus, not being able to comprehend communication nuances and not being able to see or hear the individual may cause severe interferences in communication and therefore endanger nursing quality and patient safety. This can also result in workarounds and invisible additional work, which was identified by Golay [5] as unintended consequences of healthcare information technology. This speaks for the need to further develop such services to enhance their usability, usefulness and efficiency for both patients and healthcare professionals.

Digital literacy includes a variety of core competence areas, among others “being able to connect, share, communicate and collaborate with others effectively in digital environments” [41] (p. 477). In the current study, communication was challenged by digital triage, as also seen in previous studies [42, 43]. The current findings indicate that nurses’ level of comfort, with either writing or talking, may affect their ability to use digital triage efficiently. Digital triage may thereby counteract the aim of digital health to enhance efficiency. By contrast, nurses who enjoyed working with written communication seemed more appreciative of the “changed tools”, seeing new, handy possibilities with digital triage as they described a quieter working environment. This may be a factor affecting nurses’ overall work satisfaction and, potentially, work retention [44].

Qualitative interview studies of nurses’ experiences of digital triage have not been identified in the current study. However, Schmidt et al. [40] performed a systematic review of both qualitative and quantitative articles about factors affecting communication in telephone triage. The authors reported a strong connection between communicative skills and the information obtained from HSP. Furthermore, communicative skills were predictors of establishing trust and confidence as well as HSP satisfaction. Both HSP and nurses reported that communicative skills included listening, being clear and informative, facilitating two-way communication, as well as personal traits such as being calm and empathic. Thus, for nurses not comfortable with writing, the shift of communication means from speaking to writing may endanger nurses’ ability to give safe and high-quality of care. In addition to communicative skills, knowledge, expertise and experience affect nurses’ overall performance and sense of security in telephone triage [40]. Considering the prerequisites of digital triage, including the loss of clinical ear and the demand for digital literacy, digital triage may not be an optimal task for novices, but ought to be carried out by experienced nurses. Digital literacy may therefore be of interest to explore further. Furthermore, it is important to use digital triage as a complement to telephone- and physical triage rather than as a replacement, not least with consideration taken for the risks associated with the digital divide and, e.g., patients with poor digital and health literacy [10, 45].

Video contact, however, is reported as a useful and effective tool in primary care [46]. The nurses in the current study emphasised the possibility of combining a (distant) clinical eye with the clinical ear, also entailing the possibility of synchronous communication. It may also partially counteract the negative consequences of the so-called digital divide by evading the high demand for literacy that digital triage postulates. Nonetheless, nurses were reluctant to use video services, even though they were available at some of the healthcare centres. A possible explanation may be a lack of hands-on experience [46]. Skills training for nurses in using such tools may thus be warranted to ensure efficient and safe assessments with the use of such technology in the future [8, 17].

### Strengths and limitations

A qualitative inductive design was chosen to investigate experiences of a—in this case relatively new—phenomenon aiming to get a holistic and deep understanding of the whole [26]. Interviews were chosen to collect information and gather rich and detailed data to gain insight into nurses’ perspectives on digital triage [30]. The selection of participants through the help of intermediators may represent a risk of bias, with potentially positively skewed participants, as compared to a recruitment process targeting all available personnel fulfilling the inclusion criteria. Nonetheless, the most important inclusion criteria were met, namely profession and experience of digital triage. In addition, the data were rich and included both positive and negative experiences, i.e. nuanced experiences of digital triage, indicating that the participants felt safe to share both kinds of experiences during the interviews. The same interviewer and the interview guide were used throughout all interviews facilitating homogeneous data collection, thus strengthening dependability [26].

The findings are based on a limited number of interviews, which can be argued to be a small sample to gain saturation [31]. The aim of qualitative studies is nevertheless not to generalise findings, but to examine participants' experiences in-depth to see if there are reasons to further investigate the topic [30]. The reported findings recurred in most of the interviews thus reinforcing the findings’ credibility [32]. The study only included participants from a restricted number of HSP in a specific region of Sweden, thus potentially limiting the findings’ transferability. Further studies, in additional regions and countries, are hence called for to expand on the current findings. Additionally, the different digital health services used by the nurses in the current study may also affect the findings’ transferability [32]. Four nurses used both 1177 Health Guide and online services in combination with a digital triage and chat service, whilst two of the nurses exclusively used 1177 Health Guide and online services. Nevertheless, similarities emerged among both types of services as they both encompass asynchronous written communication. The digital triage and chat service had been in use for 11 months when the nurses were interviewed, which can be considered a relatively short time. This factor ought to be taken into consideration in future studies as the current findings, although rich, are limited to the timeframe in question, potentially also limiting the transferability of the results to contexts with more or less experience of digital triage.

The interviewer (first author, E.R.) and second author (U.J.) had professional experience in primary care, telephone triage, and the digital services examined, which can be argued to represent a risk of bias. The third author (S.S.), although experienced in e-health research, did nonetheless not have professional experience from telephone/digital triage in primary care, thereby weighing up the risk of these pre-understandings influencing the analysis process and findings [26]. Nonetheless, the authors’ experiences may also represent an advantage, facilitating a deeper understanding of the phenomenon at stake and the ability to ask relevant follow-up questions [30, 32]. The interviews focused on conscious naivety to ensure openness [30], looking for a variety of experiences, both positive and negative, which also came through in the current findings. Quotes illustrate the authors’ interpretation of the empirical data, contributing to the transparency of the analysis process and strengthening the results’ trustworthiness. A thorough description of how, where and by whom as well as from whom data are collected, is provided in the methods section enabling the reader to evaluate the research process and the results’ potential transferability [22] and trustworthiness.

### Conclusion and clinical implications

Digital health is meant to improve quality, efficiency and equality. The current study aimed to explore nurses’ experiences of digital triage. The findings tell of a substantial change in nurses’ professional toolbox that demanded other skills and circumstances than they were used to working with. Due to limitations in communication and communication skills, digital triage alone may lead to an impaired workflow, quality of care and patient safety, as well as maintain the digital divide. However, it can enhance nurses’ work with the addition of (distant) eyesight, convenient communication for those who are comfortable with writing, and a gain of time for consultation and reflection. These findings, taking into account the limitations of the study, shed light especially on the importance of developed reading and writing skills, in both nurses and health-seeking individuals.

Digital triage is suggested to be used as a complement to telephone and physical triage and carried out by experienced nurses and potentially nurses specialised in digital health. Therefore, digital triage, including the potential possibilities encompassed by video triage, may be of interest to explore further, as it may have implications on both educational and clinical levels. It is also of great importance to further develop such systems and their usability to make them worthwhile for nurses and patients to use. The current study contributes insights regarding new competencies that nurses and patients must have or gain to be able to benefit from the possibilities of digitisation of primary healthcare.

## Data Availability

Original data and scheme of the analysis process are available from the corresponding author on reasonable request.
